# FOXOs and their roles in acute and chronic neurological disorders

**DOI:** 10.3389/fmolb.2025.1538472

**Published:** 2025-04-07

**Authors:** Yasin Asadi, Rozenn K. Moundounga, Anand Chakroborty, Augustina Pokokiri, Hongmin Wang

**Affiliations:** ^1^ Department of Pharmacology and Neuroscience, Texas Tech University Health Sciences Center, Lubbock, TX, United States; ^2^ Garrison Institute on Aging, Texas Tech University Health Sciences Center, Lubbock, TX, United States; ^3^ Center of Excellence for Translational Neuroscience and Therapeutics, Texas Tech University Health Sciences Center, Lubbock, TX, United States

**Keywords:** forkhead transcription factors, FOXO, neuron, injury, brain, stroke, Alzheimer’s disease, Huntington’s disease

## Abstract

The forkhead family of transcription factors of class O (FOXOs) consisting of four functionally related proteins, FOXO1, FOXO3, FOXO4, and FOXO6, are mammalian homologs of daf-16 in *Caenorhabditis elegans* and were previously identified as tumor suppressors, oxidative stress sensors, and cell survival modulators. Under normal physiological conditions, FOXO protein activities are negatively regulated by phosphorylation via the phosphoinositide 3-kinase (PI3K)-Akt pathway, a well-known cell survival pathway: Akt phosphorylates FOXOs to inactivate their transcriptional activity by relocalizing FOXOs from the nucleus to the cytoplasm for degradation. However, under oxidative stress or absent the cellular survival drive of growth factors, FOXO proteins translocate to the nucleus and upregulate a series of target genes, thereby promoting cell growth arrest and cell death and altering mitochondrial homeostasis. FOXO gene expression is also regulated by other transcriptional factors such as p53 or autoregulation by their activities and end products. Here we summarize the structure, posttranslational modifications, and translocation of FOXOs linking to their transcriptional control of cellular functions, survival, and death, emphasizing their role in regulating the cellular response to some acute insults and chronic neurological disorders. This review will conclude with a brief section on potential therapeutic interventions that can be used to modulate FOXOs’ activities when treating acute and chronic neurological disorders.

## Introduction

Transcription factors bind to DNA to regulate gene expression, and these proteins function to maintain cellular and developmental processes by activating or inactivating target genes based on the specific needs of a cell (Lehmann et al., 2003; Wen et al., 2012). Mutations in transcription factors can contribute to the progression of various diseases such as neurological disorders, developmental syndromes, diabetes, cancer, cardiovascular disease, and many others (He et al., 2023; Lee and Young, 2013). The forkhead transcription factor (FOX) family is evolutionarily conserved and divided into Fox A through S (Greer and Brunet, 2005). Among the forkhead family of transcription factors, the FOXO subgroup consists of four proteins that are considered the mammalian orthologs of *Caenorhabditis elegans* daf-16: FOXO1 (FKHR), FOXO3 (FKHRL1 or FOXO3a), FOXO4 (AFX) (Huang and Tindall, 2007). The forkhead domains were first discovered in *Drosophila*–where the forkhead gene proved crucial for embryo development. Furthermore, the forkhead gene was identified in the early embryo and determined to be involved in the regulation of the transcription of genes (Weigel et al., 1989). After identifying these proteins in *Drosophila*, there was a rapid accumulation of sequences identified by different labs, which led to the same protein having multiple names. This problem was rectified at the first International Meeting on Forkhead/Winged Helix Proteins in California in 1998 when the meeting decided that human Fox proteins would be capitalized with all letters; rodent Fox proteins would be capitalized with the first letter; and chordate Fox proteins would be capitalized with the first and subclass letter (Kaestner et al., 2000).

According to previous studies, one FOXO gene in *C. elegans* and *Drosophila* is daf-16 and dFOXO, respectively. At the same time, there are four members of the FOXO family in mammals: FOXO1 (known as forkhead in rhabdomyosarcoma, FKHR), FOXO3 (also known as FOXO3a, or forkhead in rhabdomyosarcoma like protein 1, FKHRL1), FOXO4 (also known as the acute leukemia fusion gene located in chromosome X, AFX), and FOXO6 (Webb et al., 2016). Due to their significant roles in cell survival, proliferation, differentiation, stress response, inflammation, apoptosis, and aging, FOXOs have been investigated extensively in a variety of disorders (Wang et al., 2016). This review mainly encompasses the role of FOXOs in acute and chronic neurological disorders. We will first summarize the domain structures of FOXO proteins, and then discuss the pathways leading to the inactivation or activation of FOXOs. Then, we will review the literature highlighting the role of FOXOs in some acute and chronic neurological disorders.

### FOXO proteins structures

**FIGURE 1:**
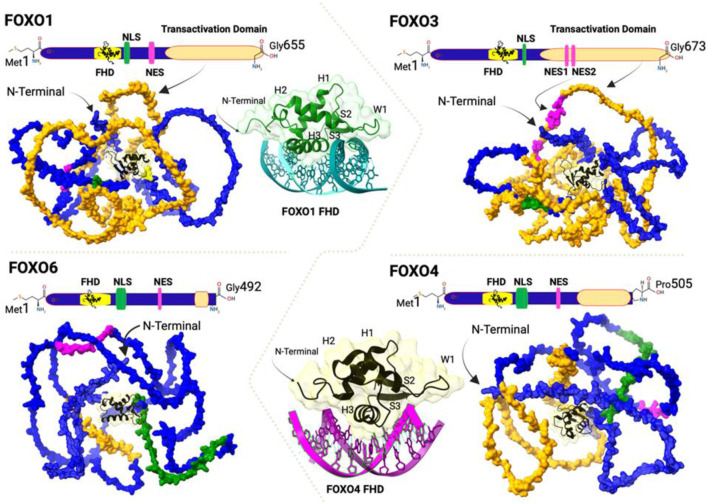
Schematic representation of the linear and three-dimensional (3D) structures of FOXO transcription factors showing Forkhead DNA-Binding Domain (FHD, yellow), Nuclear Localization Sequence (NLS, green), Nuclear Export Sequence (NES, pink), and Transactivation Domain (orange). The 3D models are generated using the Swiss Model (https://swissmodel.expasy.org) from the FOXO1 (GenBank: NP_002006.2), FOXO3 (GenBank:NP_001446.1), FOXO4 (GenBank: NP_005929.2), and FOXO6 (GenBank: NP_001278210.2). The butterfly-like appearance of the DNA binding domain is based on the crystal structure of FOXO1 DBD (green) bound to DNA in teal (PDB:3CO6) and FOXO4 DBD (black) bound to DNA in magenta (PDB: 3L2c). The respective ribbons of FOXO1 and FOXO4 visualizes Helix (H1,H2 and H3), Sheet (S1 and S2) and Wing (W1). All figures are created using the UCSF Chimera X 1.8 (https://www.cgl.ucsf.edu/chimerax/) and BioRender (https://www.biorender.com).

Mammalian cells contain four FOXO proteins that share a similar structure consisting of four domains: a highly conserved forkhead DNA-binding domain (DBD), a nuclear localization signal (NLS) downstream of DBD, a nuclear export sequence (NES), and a C-terminal transactivation domain (Figure 1). FOXO1 and FOXO3 proteins have approximately similar length amino-acid residues (655, 673 respectively), whereas FOXO4 and FOXO6 sequences are shorter (505, 492 amino acid residues, respectively). Sequence alignment analysis suggests that several regions of FOXO proteins are highly conserved, including the N-terminal region surrounding the first phosphorylation site mediated by AKT/protein kinase B (PKB), the forkhead DBD, the region containing NLS, and the region of the C-terminal transactivation domain (Figure 1). FOXO binding to promoters and enhancers of target genes is a prerequisite for the regulation of gene expression; however, they also have the capacity for such functional output without binding to target DNA directly (Ramaswamy et al., 2002).

FOXO proteins bind DNA through their conserved forkhead domain which contains three alpha-helices (H1, H2, and H3), three beta-sheets (S1, S2, and S3), and two wing-like loops (W1 and W2) (Clark et al., 1993). Despite the similarity among different FOXO-DBDs, structural studies have suggested that the secondary structure and the three-dimensional conformation of different FOXO-DBDs are different. This is supported by the high-resolution crystal structures of FOXO1, FOXO3, and FOXO4 DBDs bound to DNA duplex (Bouraet al., 2010; Brent et al., 2008; Tsai et al., 2007). For instance, although FOXO4-DBD shows a similar recognition to the core sequence (5′-AAACA-3′) as FOXO1 and FOXO3, the loop between helices H2 and H3 of its DBD has a different conformation compared to other FOXO DBDs. Moreover, the H3 of FOXO4-DBD is shorter by about one turn at the N-terminus, and the fourth short helix H4 is shifted closer to the C-terminus of helix H2 (Boura et al., 2010; Weigelt et al., 2001). On the other hand, wing W1 of FOXO4-DBD is closer to the DNA and interacts with the phosphate backbone of the target DNA (Kang et al., 2024) (Figure 1). Wing W2 is missing in the FOXO4-DBD–DNA and FOXO1-DBD–DNA structures, while the N-terminal loop is missing in the FOXO3-DBD–DNA structure (Boura et al., 2010; Brent et al., 2008; Tsai et al., 2007). These different conformations of FOXO-DBDs are likely to have biological relevance.

### Regulation of FOXO functions

In addition to DBD, all FOXO proteins contain NLS and NES which play a crucial role in the protein trafficking between the cytoplasm and nucleus (Brown and Webb, 2018). In contrast to the NLS, the NES is essential for the protein to translocate to the cytoplasm under conditions of stress (Wang et al., 2014), which is determined largely by posttranslational modifications at various sites of FOXOs. The specific posttranslational modifications discussed below alter FOXO activity by impacting their ability to bind with DNA, localization, and protein-protein interactions (Zhaoet al., 2011).

**FIGURE 2:**
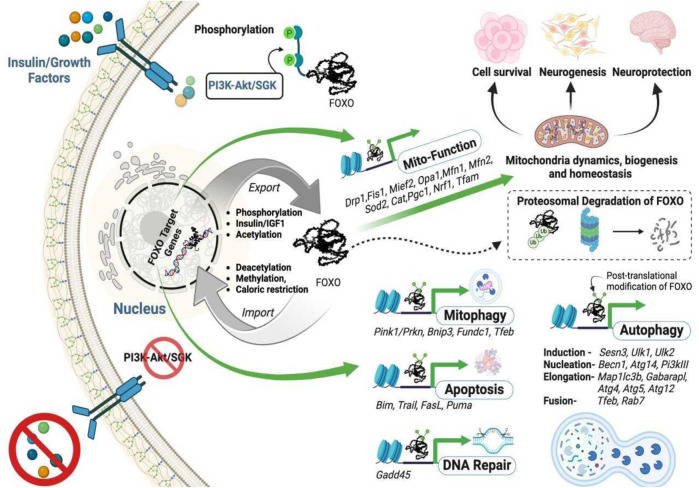
The intracellular trafficking of FOXO transcription factors and their functions. The absence of growth factor signaling, FOXO deacetylation, or FOXO methylation allows FOXOs to translocate to the nucleus to activate their targeted genes. In contrast, phosphorylation, or acetylation of FOXOs, or an increased insulin/growth factor level induces nuclear export of the proteins. The transcription (in green arrow) of selected genes is crucial for maintaining mitochondrial homeostasis by sustaining the triad mitochondrial biogenesis (*Pgc1, Nrf1, Tfam*), mitochondrial dynamics involving fusion (*Mfn1, Mfn2, Opa1*), fission (*Drp1, Fis1, Mief2*), and function, mitophagy (elimination of mitochondria through autophagy), apoptosis, and DNA repair.

Under physiological conditions, FOXO proteins are in the nucleus where they can regulate gene transcription (Brunet et al., 1999). However, FOXOs are shuttled to the cytosol and ubiquitinated during stress. Upon ubiquitination, these proteins are degraded by the ubiquitin-proteasome system, which ultimately reduces the expression of their target proteins (Farhan et al., 2017) (Figure 2). FOXO proteins can undergo modifications via various mechanisms to become activated or inactivated: They undergo inhibitory phosphorylation by protein kinases such as Akt, SGK, IKK, and CDK2 in response to external and internal stimuli which makes them inactive. On the other hand, they are activated by upstream regulators such as JNK and MST1 under stress conditions. Usually, their actions in the cell are counterbalanced by the acetylases CBP, p300, and the deacetylase SIRT1. Their activity can also be regulated by polyubiquitylation: polyubiquitination of FOXO1 and FOXO3 leads to the degradation of the proteins by the proteasome, whereas monoubiquitylation of FoxO4 facilitates its nuclear localization and augments its transcription (Huang and Tindall, 2007). Although there are many regulatory factors and pathways for FOXO activities, activation of Akt (protein kinase B) via insulin/IGF-1 signaling is a major negative regulatory pathway of FOXO activities. Akt is activated when insulin binds to its receptor (Andjelkovic et al., 1997; Brunet et al., 1999; Kops et al., 2002). This ligand-receptor interaction causes autophosphorylation via tyrosine kinase domains, which recruits adapter proteins to recruit phosphoinositol-3-kinase (PI3K). PI3K yields phosphatidylinositol triphosphate and the eventual activation of Akt, which negatively controls the activation of FOXOs by phosphorylating and inhibiting these proteins. Akt phosphorylates FOXOs on specific serine and threonine residues within their amino acid sequences (Brown and Webb, 2018). Once FOXOs are phosphorylated, they are unable to bind to DNA and therefore translocate to the cytoplasm for degradation by the proteasome via the SKPI-CUL-F-box, an E3 ligase, that specifically recognizes the phosphorylated FOXO proteins and adds ubiquitin to the protein (Huang et al., 2005). Thus, once shuttled to the cytoplasm, these proteins cannot induce the expression of their target genes. Caloric restriction is one potential strategy for controlling the phosphorylation of these proteins, which could reduce the expression of the insulin/IGF-1 pathway-regulated genes.

Acetylation of FOXO proteins modifies their DNA-binding activity. Under physiological conditions, Histone deacetylases remove acetyl groups to mediate their biological functions, and previous studies have shown that the DNA binding capabilities of these proteins are enhanced when deacetylated. However, in the presence of stress, histone acetyltransferases add acetyl groups at specific lysine sites on FOXO3 proteins, leading to the inability of FOXO proteins to regulate their target genes. In addition to phosphorylation and acetylation, FOXO proteins can also be subjected to methylation. FOXO1 is methylated by the PRMT (protein arginine methyltransferases) to inhibit Akt-mediated phosphorylation, which allows the target genes to be continually expressed (Yamagata et al., 2008). When Set9 methylates FOXO3, the transcription activity of FOXO3 proteins is enhanced (Calnan et al., 2012). Due to the variety of these proteins being readily susceptible to stress and posttranslational modifications, researchers should consider the impact each posttranslational event has on specific cell types and diseases of interest (Figure 2).

## Role of FOXOs in acute neuronal injuries

### Peripheral nerve injuries (PNIs)

PNIs involve any damage to the nerves that connect the brain and spinal cord to the rest of the body (Dong et al., 2023). 43 motor and sensory peripheral nerves control many essential functions in the body and serve as direct communication lines between the central nervous system and the rest of the body. As of 2021, it was reported that 13 to 23 people per 100,000 typically suffer from PNI, with most cases, if not all, resulting in lifelong disability (Zhang et al., 2022). In response to an acute sciatic nerve crush, the FOXO3 protein level in the neurons and glia of the lumbar dorsal root ganglia was reduced 24 h after injury, which was associated with downregulation of FOXO3 and p27^kip1^ protein level (Wang et al., 2009). Another study, also using a sciatic nerve model, showed decreased FOXO3 levels during the early stages of the injury, followed by an increase in FOXO3 phosphorylation. They concluded that FOXO3 is involved in Schwann cell differentiation through the PI3K/Akt pathway, which may play an important role in nerve regeneration after injury (Zhao et al., 2011). However, the potential mechanism of FOXO3 in axonal regeneration following sciatic nerve injury remains unknown.

### Spinal cord injury (SCI)

SCI refers to any damage to the spinal cord that plays a crucial role in connecting the brain to the rest of the body. Such an injury often results in partial or total loss of function in the body parts affected by the damage. Worldwide, more than 15 million people live with spinal cord injuries, and for most of them, the quality of life is low, with higher mortality rates (Adegeest et al., 2024). In experimental SCI, the FOXO3 overexpression showed a proliferative effect via an increase in p27kip1 protein and inhibited TNF-α protein production (Lu et al., 2018). Tetrahydrocurcumin the most active component of curcumin has shown a decrease in inflammatory factors and oxidative stress by enhancing the expression of FOXO4 in the SCI model in rat (Xi et al., 2019).

### Traumatic brain injury (TBI)

According to the Centers for Disease Control, in 2021, there were 69,473 reported deaths related to TBI, not including many unreported cases. This translates to roughly 190 TBI-related deaths per day in the United States. TBI encompasses any physical damage to the brain that can result in various impairments. It ranks as one of the most common causes of disability in adults and can vary from something as simple as a mild concussion to something as serious as coma or death. FOXO proteins play a crucial role in the nervous system and encounter pathologic conditions. After TBI, FOXO protein expressions upregulate in the human brain (Liu et al., 2021). A recent study by Maiese et al. demonstrated that FOXO3a is a diagnostic factor for TBI and the increase in FOXO3a in tissue level positively correlates with apoptosis and autophagia (Maiese et al., 2023). Similarly, TBI also increases FOXO1, FOXO3, and FOXO4 protein levels in mouse brains, with a peak elevation at 24 h following the incident (Liu et al., 2021). The similar results were also observed from another study that showed that TBI significantly increases FOXO3 levels in the serum of patients, as well as in experimental animals induced by a weight-drop device (Sun et al., 2018). Moreover, TBI induces the translocation of FOXO3 from the cytosol to the nucleus, while knockdown of FOXO3 attenuates TBI-caused neurobehavioral deficits and neuronal injury in the hippocampus. Interestingly, nuclear accumulation of FOXO3 is associated with a high level of autophagy pathway gene expressions, and silencing FOXO3 remarkably inhibits the induction of neuronal autophagy after TBI (Sun et al., 2018), suggesting that FOXO3 facilitates TBI-induced brain injury via the overactivation of neuronal autophagy. According to these studies, it seems that the inhibition of FOXO3 could be a therapeutic strategy for TBI.

### Ischemic stroke

An ischemic stroke occurs when the blood flow is blocked in some part of the brin, resulting in cerebral ischemia. Stroke is the second leading cause of death and the third leading cause of disability worldwide (Prust et al., 2024). As transcription factors, FOXOs play a crucial role in cellular processes due to their ability to activate or suppress the activity of their target genes. These cellular processes include the cell cycle, apoptosis, proliferation, differentiation, inflammation, autophagy, metabolism, and oxidative stress (Farhan et al., 2017; Wang et al., 2014). Among them, oxidative stress, inflammation, and apoptosis have a key role in stroke.

Among the four members of FOXO proteins, FOXO1, FOXO3, and FOXO4 are ubiquitously expressed, while FOXO6 is specifically expressed in the liver, skeletal muscle, and the hippocampus of the brain (Biggs et al., 2001; Huang and Tindall, 2007). Often found to be co-expressed in the same cell type, the three FOXO members, FOXO1, FOXO3, and FOXO4, have significant function redundancies that might be a safeguard against the accidental loss of FOXO function, which could otherwise lead to cancer development (Schmitt-Ney, 2020). Ischemic pre-conditioning upregulates Akt activity, leading to FOXO inhibition and promotion of neuronal survival against a subsequence severe ischemic insult (Zhan et al., 2010). Evidence suggests that SIRT1 activation may be dependent on PI3K/AKT signaling while SIRT2 inhibitors AK1 and AGK2 have demonstrated the capacity to attenuate the p-AKT/Foxo3a signaling axis in ischemic stroke models (Duan et al., 2019). However, despite highly structural and functional similarities, previous data have suggested that the physiological roles of FOXOs appear functionally diverse in mammals (Hosaka et al., 2004; Wang et al., 2009). A better understanding of the biological function of FOXOs in ischemic stroke-caused cell death would aid in designing effective therapeutics for treating the disorder.

Among the four FOXO members, FOXO4 appears to play a different role from other FOXOs, as the loss of FOXO4 does not cause any notable changes in the mouse. In contrast, FOXO1 deficiency is embryonically lethal, while FOXO3 deficiency exhibits age-dependent infertility in female mice (Castrillon et al., 2003; Hosaka et al., 2004). Following cardiac ischemia, FOXO4 enhances the interaction of leukocytes with the endothelial cells in blood vessels to promote early tissue inflammation (Zhu et al., 2015). In contrast, downregulation of FOXO4 suppresses oxidative stress-induced cell death in proangiogenic cells and promotes neovascularization in ischemic limbs (Nakayoshi et al., 2014). Investigation of the role FOXO4 plays in ischemic stroke-induced brain injury and dysfunction may lead to developing novel therapeutic strategies and agents for treating stroke-caused disorders. Administration of compounds with antioxidant and anti-inflammatory effects alleviated nerve damage by upregulating Sirt1/FOXO3 and inactivating the NF-κB pathway in mice models of cerebral ischemia (Zhang et al., 2022).

After cerebral ischemia-reperfusion, reactive oxygen species and apoptosis increase and superoxide dismutase activity decreases. These changes are accompanied by the Sirt1 downregulation and FOXO1 upregulation in the *in vivo* and *in vitro* models of ischemia-reperfusion (Meina et al., 2024). Deletion of the FOXO4 gene in vascular endothelial cells led to an increase in cell viability through a decrease in apoptosis (Cui et al., 2024).

Another interesting study has demonstrated that the expression levels of SIRT1, FOXO3, CAT, BRCC3, and NLRP3 in the hippocampus of a stroke model are significantly elevated following the transcutaneous electrical acupoint stimulation (TEAS) intervention. A SIRT1 inhibitor was shown to attenuate the anti-oxidative and anti-neuroinflammatory roles of TEAS and reverse the TEAS-mediated elevation of SIRT1 and FOXO3 after the intervention (Tan et al., 2024). These studies suggest that FOXOs involve in stroke-induced brain injury and manipulation of their activities could be therapeutic interventions.

## Role of FOXOs in chronic neurological disorders

The prevalence of neurodegenerative disorders is projected to increase as the age of the population increases (Mekkes et al., 2024). In the United States alone, the number of people over the age of 65 is projected to increase to 88 million, which will increase the number of individuals suffering from a neurodegenerative disorder (Alzheimer’s Association, 2018). As FOXOs play a crucial role in modulating aging, cell death and survival, neuroinflammation, and neuronal physiological functions, it is imperative to understand their expressions and functions in neurodegenerative disorders.

Different FOXOs appear to have distinct expressions in different brain regions: FOXO1 is found in the ventral and posterior regions of the hippocampus, highlighting the role of these FOXO proteins in memory. FOXO3 is abundant in the cortex and cerebellum, indicating that this protein may play an important role in cognitive and motor functions. FOXO6 expression is predominately found in the nucleus accumbens, which could suggest its role in the reward pathway, cognitive function, and emotion (Hoekman et al., 2006; Maiese et al., 2009). The mapping of FOXOs within the brain could provide further insight into the implications of these proteins in neurodegenerative disorders. Cognition impairment is a pathological condition reviled in neurodegenerative diseases (Qin et al., 2024). FOXO3 regulates neuronal dynamics, in this regard, FOXO3 could modulate the onset or amelioration of cognitive impairment in many neurological disorders. Based on the preliminary clinical study data, FOXO3 is a potential therapeutic target for treating cognitive impairment-related neurodegenerative diseases (Liu et al., 2024).

### Huntington’s disease (HD)

HD is a neurodegenerative disorder caused by an extension of CAG trinucleotide repeats within the Huntingtin gene (Nopoulos, 2016). Many laboratories, including ours, have focused on understanding the role of FOXOs in this disorder. FOXO3 is upregulated and is found predominantly in the nucleus in HD cells (Santo and Paik, 2018); however, the underlying mechanism is unknown. One study has shown that the mRNA level of FOXO3 increased in the caudate and cerebral cortex of *postmortem* HD patients in comparison to non-diseased control brain samples (Kannike et al., 2014). Studies from our lab on HD utilizing HD-iPSC-derived neurons exhibited diminished expression of FOXO4 and proteasome activity compared to WT-iPSC-derived neurons. Additionally, inhibiting Akt showed an upregulation in FOXO4 levels and increased proteasome levels, which supports the hypothesis that phosphorylation of FOXO4 alters the effects of the downstream gene targets of FOXOs (Liu et al., 2017). In reprogrammed neural stem cells, FOXO3 can repress cell senescence, antagonizing p16INK4a expression via repression of the transcriptional modulator ETS2. The result of this study suggests that cellular senescence may develop during neuronal differentiation in HD and that the FOXO3-ETS2-p16INK4a axis may be part of molecular responses aimed at reducing or preventing this from occurring (Voisin et al., 2020). Interestingly, studies related to FOXO1 and FOXO6 in HD are currently lacking.

### Parkinson’s disease (PD)

PD is a neurodegenerative disorder defined by substantial motor dysfunctions due to the loss of dopaminergic neurons in the midbrain (DeMaagd and Philip, 2015). Expression analysis of Parkinson’s disease brain samples in the prefrontal cortex has revealed that FOXO1 expression is significantly enhanced (Dumitriu et al., 2012). Immunohistochemistry analysis revealed upregulation of FOXO3 in the dopaminergic neurons in the substantia nigra par compacta region of the rat brain, and the constitutive activation of FOXO3 using viral vectors leads to neuronal loss in this brain region. Co-injection of the viral vector that increases FOXO3 activity together with a viral vector encoding α-synuclein showed protection in the dopaminergic neurons (Pino et al., 2014). In this context, FOXO3 has either a positive or a negative effect depending on the function status, cell type, and severity of the stress stimulus: while constitutive activation has proapoptotic effects leading to neuronal loss, inhibition of FOXO-mediated transcription by a dominant-negative competitor causes oxidative damage and is detrimental at high vector dose in neurons. This suggests that dopaminergic neurons are particularly vulnerable to changes in FOXO3 activity in the substantia nigra, according to Pino et al. Furthermore, exposure to manganese causes patients to exhibit clinical symptoms that resemble PD. The administration of manganese showed an enhanced expression of phosphorylated FOXO levels, which highlights the inability of these proteins to regulate their target genes due to phosphorylation upon administration of manganese (Exil et al., 2014). Additional studies on the role of FOXO4 and FOXO6 in PD are crucial to provide a clearer picture of the effect of these proteins on PD. Olfactory dysfunction is one of the adverse effects that occurs frequently in PD, while nicotine ameliorates the olfactory dysfunction by recruiting the prok2R/Akt/FOXO3 signaling pathway (Guo et al., 2024).

### Alzheimer’s disease (AD)

AD is a neurodegenerative disorder and is expected to impact over 7 million Americans over the age of 65 by the year 2050 (Alzheimer’s Association, 2018). To determine the effect of the insulin/IGF signaling pathway on AD, an AD mouse model with decreased IGF-1R signaling was generated by crossing the AβPPswe/PSEN1dE9 mice with *Igf1r* ^+/−^ mice. Behavioral tests conducted on these mice showed a reduction in learning and memory deficits, which the authors attributed to Aβ forming into larger fibrillar structures to decrease their toxicity. In regards to this study, the authors did not specifically examine the expression and activity of FOXOs in the brains of these mice; however, based on observations made in *C. elegans* (Cohen et al., 2009).

**FIGURE 3:**
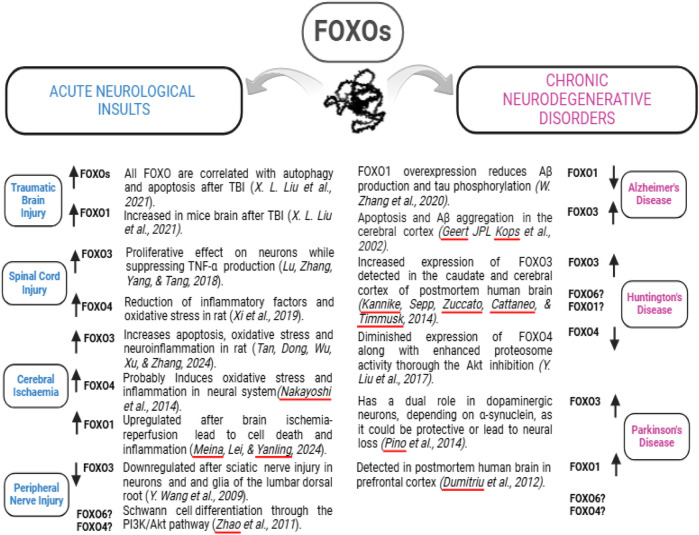
Altered FOXO expression induced by different neurological disorders. A specific neurological disorder may up- or downregulate the expression of a FOXO protein, indicated by the upward or downward arrows. The related literature is listed in italics. The question marks indicate unknown alterations.

PTEN-induced kinase 1 (Pink1) and Parkin RBR E3 ubiquitin-protein ligase (PARKIN or Prkn) genes play an important role in mitophagy, mitochondrial motility, and maintaining mitochondrial size; in contrast, dysregulation of PINK1/PARKIN signaling was seen in a variety of neurodegenerative diseases, including AD and PD (Ivankovic et al., 2016; Kitada et al., 1998; Ozgenet al., 2022; Quinn et al., 2020; Valente et al., 2004; Ye et al., 2015). Among the FOXO protein family, FOXO3 plays a crucial role in mediating cellular stress responses, including hypoxia, DNA damage, oxidative stress, and caloric restriction (Kops et al., 2002). Overexpression of FOXO3 has been linked to neurodegeneration in AD and its inhibition has led to the alleviation of cell apoptosis and Aβ aggregation (Kops et al., 2002). In the AD condition, Aβ inhibits the Akt signaling pathway (Choi et al., 2012; Gabbouj et al., 2019; Kitagishi et al., 2014; Lee et al., 2009), thereby activating FOXOs and leading to neurodegeneration (Manolopoulos et al., 2010; Sanphui and Biswas, 2013; Shi et al., 2016). In contrast, an *in vitro* study showed that FoxO1 overexpression reduces Aβ production and tau phosphorylation, suggesting that FOXO1 could be a novel target for AD treatment (Zhang et al., 2020). Therefore, depending on specific conditions, different FOXOs may play distinct roles. Additionally, the level of FOXO3 in the serum may be a novel blood marker for early detection of AD in the geriatric population (Pradhan et al., 2020). The conflicting reports on the activity of specific FOXO proteins may be caused by the use of distinct types of organs, cells, and existing pathological conditions (Asadi and Wang, 2024). For instance, an acute condition, such as TBI, SCI, or acute cerebral ischemia, usually upregulates the level of a FOXO protein (except FOXO3 in the condition of PNI), while a chronic neurological disorder, including AD, HD, and PD, could increase or decrease a FOXO protein (Figure 3).

## FOXOs as potential therapeutic targets

Therapeutic strategies that target FOXOs may help prevent or enhance their phosphorylation and subsequent inactivation or activation of their target genes. Therefore, identifying therapeutics that disrupt or downregulate the insulin/IGF-1 signaling pathway via small molecular compounds could be an attractive strategy for treating some neurological conditions.

Resveratrol is a chemopreventive agent utilized in cancer patients. Resveratrol decreases cellular events associated with carcinogenesis, and as a result, resveratrol acts on the insulin/IGF-1 signaling pathway to inhibit the cytoplasmic phosphorylation of FOXO3 to increase the targeting of FOXOs in the nucleus in cell cells. Additionally, resveratrol inhibits cellular migration (Srivastava et al., 2010). In a gerbil model of ischemia, intraperitoneal injections of resveratrol suppressed glial cell activation following ischemic insult (Wang et al., 2002). A study done in patients with mild-to-moderate dementia found that resveratrol is safe and well-tolerated and that it can decrease plasma Aβ40 levels in the patients. However, the treatment appeared not to improve patients’ cognitive functions (Sawda et al., 2017). Additional studies are necessary to determine the effect of different dosages of resveratrol on neurodegenerative disorders. Moreover, extended research is needed to assess the cognitive benefits of resveratrol in individuals with dementia for a more extended period.

Calorie restriction is another strategy to modulate the activity of FOXOs due to their role in the insulin/IGF signaling pathway. After an individual consumes a meal, this pathway is activated due to insulin release and binding to its receptor to decrease the amount of glucose in circulation. As a result, several studies have identified the impact of caloric restriction on aging and FOXOs. In one particular study, male FOXO3^+/−^ and FOXO3^−/−^ mice were subjected to dietary restriction; however, these genetically modified mice did not show altered lifespan in comparison to the wild-type group. However, it is noted that animals subjected to dietary restriction exhibited a decrease in tumor formation by the end of the study in comparison to the mice that had free access to food (Shimokawa et al., 2015). Another study identified that every-other-day feeding was not beneficial when behavioral effects associated with AD were tested in a 5xFAD mouse model (Lazic et al., 2020). Calorie restriction has been extensively studied in the aging process; however, the role of calorie restriction in other disorders and its impact on FOXOs in these disorders are under-studied.

It is noted that in addition to the extracellular signals induced by the insulin and IGF, FOXO activities are also regulated by other exogenous factors such as BDNF and glutamate, as well as intracellular molecules such as AMPK, sirtuin-1 (Sirt1), and JNK, which play roles in neurodegenerative diseases. For instance, Cilostazol (CLZ), a phosphodiesterase III inhibitor that is used in peripheral vascular disorders, was shown to modulate the downregulation of p65 NF-κB and Sirt1 enhancement to reduce active caspase-1 and interleukin-1β, indicative of inflammation, by enhancing glutathione via FOXO3 reduction which is believed to be the cause of CLZ antioxidant capacity. Additionally, CLZ triggered neuronal survival by promoting the phosphorylation of Akt, TrkB, and CREB and upregulation of BDNF. Therefore, these exogenous factors could be potential therapeutic candidates in neurological disorders because, in pathological conditions, insulin and IGF may not necessarily be the most prominent regulators of FOXO (Tan et al., 2024).

## FOXO inhibitors

Due to their important role in cell survival and death, manipulation of FOXO activity through an inhibitor may result in beneficial therapeutic effects on neural cells, depending on a specific disease condition. Over the past decade, many small molecule and peptidic FOXO inhibitors have been identified. In a recent study, Gupta et al. identified Fraxin as a FOXO1 inhibitor using structural biological approaches combined with molecular docking and molecular dynamics simulation (Gupta et al., 2024). However, the role of Fraxin in neurological disorders has not been tested. S1842856 is another small molecule FOXO1 inhibitor (Nagashima et al., 2010) that has been tested in many cell lines (Zou et al., 2014). It was found that AS1842856 inhibits the GSK3α/β to suppress tauopathy by accelerating GSK3α/β exocytosis in cultured neural cells (He et al., 2025), whereas the *in vivo* studies of the inhibitor in the context of a neurological disorder are still lacking. Syringin is one naturally occurring compound that exserts neuroprotective effects by phosphorylation/inhibition of FOXO3/NF-κB pathways, thereby reducing neuroinflammation after cerebral ischemia (Tan et al., 2021). In addition, microRNAs provide another type of inhibitor of FOXOs. For example, one study has demonstrated that both mRNA and protein levels of FOXO3 are significantly upregulated, while two homologous microRNAs, miR-132 and miR-212, are downregulated in human AD brains. The miR-132/212 suppress PTEN/FOXO3 signaling pathway, contributing to AD neurodegeneration (Wong et al., 2013). A definite FOXO6 inhibitor is not yet characterized but *in silico* studies have demonstrated the porfimer sodium to be a potential FOXO6 inhibitor that may suppress gastric cancer progression (Poleboyinaet al., 2023) (Table 1).

**TABLE 1:** Some identified inhibitors of FOXOs that have been tested in neurological disorders.

FOXOs	Inhibitor	Properties	Mechanism	Outcome	References
FOXO1	Fraxin	Organic compound regarded as a glucoside	Based on drug design and molecular docking analysis		Gupta et al. (2024)
	AS1842856	Small molecule	Directly binds to unphosphorylated FOXO1 protein to block transcriptional regulation	Induces pro-apoptotic genes in cancer cells	Zou et al. (2014)
	AS1842856	Small molecule	AS1842856 inhibits GSK3α/β against Tauopathy by accelerating GSK3α/β exocytosis from the neural cells	Improve cognition capacity in mice	He et al. (2024)
FOXO3	Syringin	Natural chemical compound (phenylpropanoid glycoside)	Phosphorylation/inhibition of FOXO3/NF-ΚB pathway. Decreases inflammation after ischemic stroke.	Neuroprotective effects	Tan et al. (2021)
FOXO4	FOXO4-DRI and ES2	Peptides	Disrupting the FOXO4-p53 interaction	Selective induction of apoptosis in senescent cells	Baar et al. (2017) and Le et al. (2021)
FOXO6	Porfimer sodium?	Photosensitizer used in photodynamic therapy and radiation therapy	??	??	Poleboyina et al. (2023)

To maintain cellular homeostasis, FOXO4 interacts with p53 and mediates cell senescence (Bourgeois and Madl, 2018). The FOXO4-p53 axis garnered significant attention for the discovery of senotherapeutics (Baar et al., 2017; Le et al., 2021; Tripathi et al., 2021; Zhang et al., 2023). FOXO4-D-retro-inverso (FOXO4-DRI) is a senolytic peptide that targets tumor suppressor p53 resulting in p53/p21CIP1-dependent apoptosis in senescent cells (Baar et al., 2017). One identified peptide, named ES2, disrupts FOXO4-p53 interaction by binding to the conserved region 3(CR3) of FOXO4 to activate p53-mediated apoptosis (Le et al., 2021) (Table 1). CPP-CAND is another potential senolytic candidate targeting the FHD domain of FOXO4 to interfere with the FOXO4-p53 interaction (Kang et al., 2024). Nevertheless, the clinical benefits of these senolytic peptides in treating age-related neuropathological diseases are yet to be determined.

## Conclusion

FOXO transcription factors translate environmental stimuli (including insulin, growth factors, nutritional changes, and oxidative stress) into specific gene expression programs. FOXO proteins are regulated at the protein level, but there is limited data about the factors affecting their gene expression. The functional capacity of these proteins varies depending on pathological conditions. Evidence shows the change in FOXO protein levels occurs in acute neural injuries such as acute sciatic nerve injury, ischemic stroke, PNI, and TBI; however, a detailed understanding of the factors and signaling pathways that trigger altered FOXOs is still vague. Similarly, a dearth of knowledge exists in the field of chronic neurodegenerative diseases in terms of the role of FOXOs, even though some evidence supports that FOXOs could be therapeutic targets and valuable biomarkers for the treatment and diagnosis of chronic neurodegenerative disease patients.

## Future directions

In the future, it would be beneficial for researchers to determine at what point altered FOXO proteins become implicated in disease, which could serve as a potential biomarker for the diagnosis of specific neurodegenerative diseases. Furthermore, future studies are necessary to elucidate the specific activity of FOXOs necessary to contribute to a neurological disease, which could further aid in developing therapeutic strategies for treating the diseases. Studies are needed to address the effects of specific genetically modified mice with altered FOXO proteins as well as the impact of FOXO6 in neurodegenerative disorders due to the lack of studies on this protein in these disorders. It is also important to identify any age-associated alterations in these proteins to provide evidence of their role in neurodegenerative diseases. Finally, it would be beneficial to combine cell and animal studies with *postmortem* brain sample studies where the individual suffered from a specific neurodegenerative disease.
